# Pterostilbene fluorescent probes as potential tools for targeting neurodegeneration in biological applications

**DOI:** 10.1080/14756366.2022.2091556

**Published:** 2022-06-27

**Authors:** Lidia Ciccone, Susanna Nencetti, Maria Marino, Chiara Battocchio, Giovanna Iucci, Iole Venditti, Martina Marsotto, Emiliano Montalesi, Simone Socci, Beatrice Bargagna, Elisabetta Orlandini

**Affiliations:** a Department of Pharmacy, University of Pisa, Pisa, Italy; b CISUP – Centre for Instrumentation Sharing, University of Pisa, Pisa, Italy; c Department of Science, University Roma Tre, Rome, Italy; d Department of Earth Science, University of Pisa, Pisa, Italy; e Research Centre E. Piaggio, University of Pisa, Pisa, Italy

**Keywords:** Ptb fluorescent probes, spectrophotometric, pterostilbene 7-nitrobenzofurazan (NBD) derivative, pterostilbene rhodamine-B-isothiocyanate (Rhd B-Itc) derivative, citotoxicity

## Abstract

Several epidemiological studies suggest that a diet rich in fruit and vegetables reduces the incidence of neurodegenerative diseases. Resveratrol (Res) and its dimethylated metabolite, pterostibene (Ptb), have been largely studied for their neuroprotective action. The clinical use of Res is limited because of its rapid metabolism and its poor bioavailability. Ptb with two methoxy groups and one hydroxyl group has a good membrane permeability, metabolic stability and higher *in vivo* bioavailability in comparison with Res. The metabolism and pharmacokinetics of Ptb are still sparse, probably due to the lack of tools that allow following the Ptb destiny both in living cells and *in vivo*. In this contest, we propose two Ptb fluorescent derivatives where Ptb has been functionalised by benzofurazan and rhodamine-B-isothiocyanate, compounds **1** and **2**, respectively. Here, we report the synthesis, the optical and structural characterisation of **1** and **2**, and, their putative cytotoxicity in two different cell lines.

## 1. Introduction

Neurodegenerative diseases such as Parkinson's (PD), Alzheimer's AD, Amyotrophic Lateral Sclerosis (ALS), and Macular Degeneration (MS) are considered the greatest challenge for all researchers in the twenty-first century. It has been hypothesised that, in 2030, the incidence of only AD, in the world population, will be around 100 million individuals, with a very high emotional and economic cost^1^^,^^2^.

Unfortunately, the treatments available against these pathologies are not effective and, in most cases cannot prevent the onset of the disease^3–6^. Only in some cases, they allow the slowing of pathology progression guaranteeing, for short periods, an acceptable quality of life. On 7 June 2021, the Food and Drug Administration (FDA) approved Aduhelm (aducanumab) a human IgG1 anti-Aβ monoclonal antibody specific for β-amyloid oligomers and fibrils for the treatment of AD^7^, although, the scientific community has still conflicting opinions^8^^,^^9^.

Therefore, it appears very important to focus the attention on the research to prevent or to slow down the initial steps of the neurodegeneration process. The discovery of promising agents against neurodegeneration with little or no toxicity represents an important goal for all people suffering from this debilitating disease and their caregivers.

**Figure 1.**
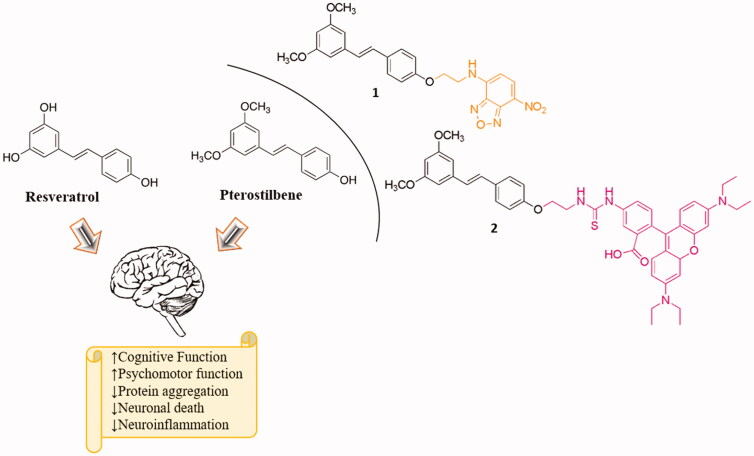
Chemical structures of resveratrol (Res) and pterostilbene (Ptb) and their positive effects in the brain. On the right side the structure of Ptb factionalised on phenolic ring with benzofurazan **1** (yellow) and with rhodamine B-isothiocyanate **2** (pink), respectively.

Recently, several studies have confirmed that plant derivate natural substances, whether of terrestrial or marine origin can reduce the incidence of different pathologies including neurodegenerative^10–14^, cancer and heart diseases^15^^,^^16^, chronic inflammation and arthritis^17^^,^^18^. Among several promising compounds, resveratrol (Res) is one of the most studied and best known for its potential as a therapeutic agent for neurodegenerative diseases^19–21^. Res is a polyphenol (trans-3,4,5–trihydroxystilbene), Figure 1, found in several plants, grapes, blueberries raspberries and peanuts. Several *in vitro* and *in vivo* studies^22–25^ report that Res is a promising compound for the prevention and the treatment of the neurodegeneration. In particular, in a randomised double-blind placebo-controlled trial versus AD reported that Res is well-tolerated and safe for the patients^26^.

However, the clinical use of Res is limited because of its rapid metabolism and its poor bioavailability, encouraging researchers to derivatize this stilbene to overcome its limit^27^. In parallel, new polyphenols were studied for their neuroprotective effects. In particular, the researches have turned attention on the polyphenol pterostilbene (Ptb) (trans 4-(3,5-dimethoxystyryl)phenol); the dimethyl analogue of Res (Figure 1) found among other foods, in grapes, blueberries and almonds^28^^,^^29^.

Studies show that, following oral administration, Ptb has an approximately 4-fold greater (about 80% versus 20%) bioavailability and 7.5-fold (105 min versus 14 min) longer half-life if compared to Res^30^^,^^31^. As a whole, compared with Res, Ptb seems to have a better membrane permeability and metabolic stability, which subsequently could increase its bioavailability and pharmacokinetic profile^32^.

Recent studies have demonstrated that Ptb has anti-inflammatory, anti-carcinogenic and anti-diabetic and neuroprotective properties together with a very low toxicity in animal and human models^29^^,^^33–38^. The low molecular weight and good liposolubility allow Ptb to pass through the blood–brain barrier improving neurological function after ischaemia reperfusion, exerting a neuroprotective effect^39^. Moreover, Ptb mediates neuroprotective activity mainly *via* the subtype α of oestrogen receptor (ER-α) and may be a new neuroprotective agent for nerve injury treatment and prevention^40^.

However, the metabolism and pharmacokinetics of Ptb are still scarce probably due to the lack of tools that allow following the Ptb destiny both in living cells and *in vivo*^32^.

Here, new fluorescent derivatives of Ptb **1** and **2** (Figure 1) have been synthesised and characterised to highlight their fluorescent properties *in vitro* and to evaluate their putative cytotoxicity in two different cell lines. Compound **1** possesses benzofurazan (2,1,3-benzoxadiazole, NBD) as fluorescent moiety while compound **2** is functionalised with rhodamine-B-isothiocyanate (Rhd B-Itc) (Figure 1). The use of fluorescent probes could be useful in the evaluation of the molecular pathways involved in neuroprotection process.

## 2. Result and discussion

### 2.1. Chemistry experimental procedures

**Scheme 1.**
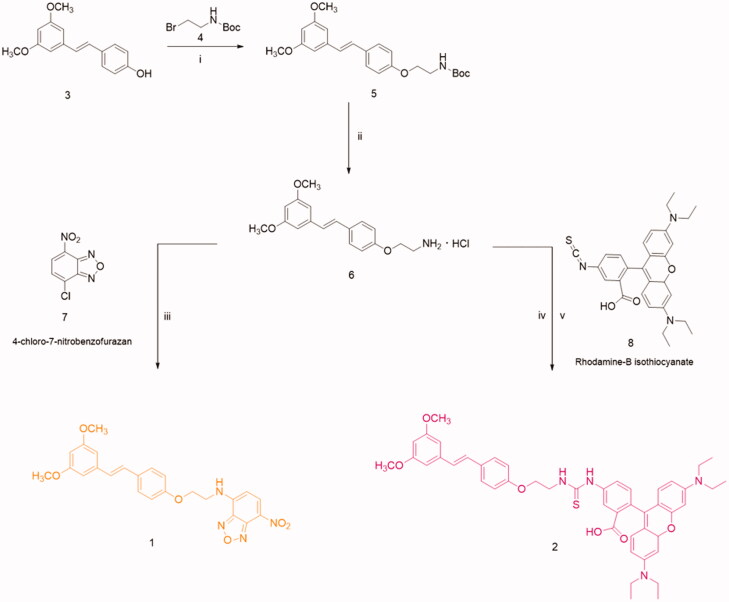
(i) K_2_CO_3_, anhydrous DMF, 50 °C, 8 h, then r.t., 36 h. (ii) CF_3_COOH, DCM, r.t., 12 h, Et_2_O • HCl, 0 °C. (iii) LiOH, NDB H_2_O/THF, r.t., 12 h. (iv) NaOH 0.1 M. (v) DMF, Rhodamine-B Itc, r.t, 48 h.

Compounds **1** and **2** were synthesised as reported in Scheme 1, following the synthetic route reported in the literature for Res fluorescent compounds^41^.

In detail, compounds **1** and **2** were synthesised *via* amine derivative **6** that was obtained from the reaction of Ptb with *N*-Boc-bromoethylamine, and subsequent deprotection of the Boc group in acidic conditions. After, the conjugation of compound **6** with benzofurazan (2,1,3-benzoxadiazole, NBD) or rhodamine-B-isothiocyanate (Rhd B-Itc) afforded the desired fluorescent pterostilbene derivatives **1** and **2**.

For compound **1**, the NBD was chosen as fluorescent probe skeleton because the NBD fluorescent derivatives possess long excitation and emission wavelengths (e.g. 464 nm, em. 512 nm) that avoid interference due to biomatrices as reported in the literature^42^^,^^43^. While, compound **2** was functionalised with Rhd B-Itc; a moiety present in numerous fluorescent compounds used to perform biological tests^44^.

### 2.2. Optical and structural characterizations

UV–visible absorption and emission characterisations were carried out on NBD, Res, **1** and **2** compounds. For all samples, absorption spectra were collected in DMSO solutions prepared at three different concentrations, and the respective calibration lines were obtained by applying a linear fitting procedure (origin included), as to calculate the molar extinction coefficient (*ε*). Maximum absorption wavelengths and *ε* values are summarised in Table 1.

**Table 1.** Maximum absorption wavelengths and ε values of NBD, Res, **1** and **2**.

Sample	*λ*_max_ (nm)	*ε* (cm^−1^ M^−1^)
**NBD**	469	7358
**Res**	310^a^	44,307
**1**	325	48,574
**2**	280	31,401

^a^As expected from literature^45^ Res absorption spectrum shows two main peaks at 310 nm and at 319 nm, respectively.

Emission spectra were also collected by exciting the four samples DMSO solutions at the maximum absorption wavelength. Excitation wavelength (*λ*_exc_), emission wavelength (*λ*_emis_), and emission intensity (*I*_emis_) values are summarised in Table 2. All absorption and emission spectra and linear fits are reported in the Supporting material (Figures S1–S5), as well as absorption wavelengths, absorbance values, emission wavelengths, and emission intensity values for each measured solution (Tables S1–S5).

**Table 2.** Excitation wavelength, emission wavelength and emission intensity (a.u.) values of NBD, Res, **1** and **2.**

Sample	*λ*_exc_ (nm)	*λ*_emis_ (nm)	*I*_emis_ (a.u.)
**NBD**	469	545	271
**Res**	325	397^a^	53
**1**	458	537	19
**2**	319	575	271

^a^Emission spectrum of Res shows one main peak at 397 nm, as expected from the literature^45^.

**Figure 2.**
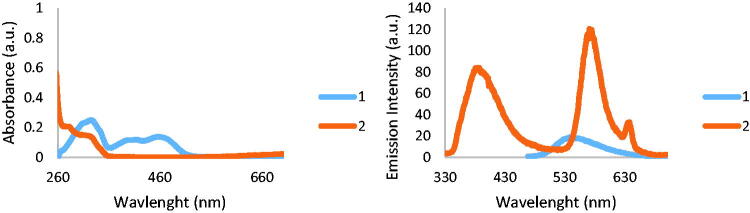
Absorption and emission spectra collected on **1** and **2**.

The absorption and emission spectra collected on **1** and **2** are shown in Figure 2.

**Figure 3.**
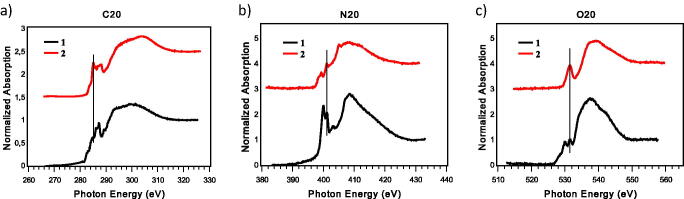
Carbon K-edge spectrum (a), nitrogen K-edge spectrum (b), and oxygens K-edge spectrum (c) of samples **1** and **2**.

The proposed molecular structure of samples **1** and **2** was also assessed in solid state by Near-Edge X-ray Absorption Fine Structure spectroscopy (NEXAFS). NEXAFS spectra are reported in Figure 3(a–c).

C K-edge spectra (Figure 3(a)) present a feature, fixed at 285.0 eV, assigned to C 1 s(C-H) → π* aromatic C = C transition; the features at 287.3 eV in **1** and at 287.0 eV in **2** are assigned to C 1 s → π* C = N/O resonance. In spectrum **1**, the features at 286.2 eV is due to C 1 s(C-R) → π* C = C transition. In spectrum **2** the feature at 288.0 eV is associated to C 1 s → π* C = S transition^46^.

In N K-edge spectra (Figure 3(b)), the feature, fixed at 401.0 eV, is due to the 1 s → π* transition of the C_ar_-N bond. The previous peaks, at 400 eV in **1** and at 398.6 eV in **2**, are assigned to N 1 s → π* C = N transition of the oxadiazole ring and to S-C = N 1 s → π* transition^46^, respectively. Finally, the two broad bands around 406 and 413 eV are assigned to N 1 s→σ* N − H and N 1 s→σ* N − C resonances, respectively.

In the O K-edge spectra (Figure 3(c)) there is a sharp peak fixed at 531.5 eV, assigned to the transition 1 s → π* of carbonyl C = O. The other feature at 530.0 eV of the black spectrum is the result of the superimposition of 1 s → π* transitions of oxadiazole oxygen and nitro group oxygens^27^^,^^47^.

**Figure 4.**
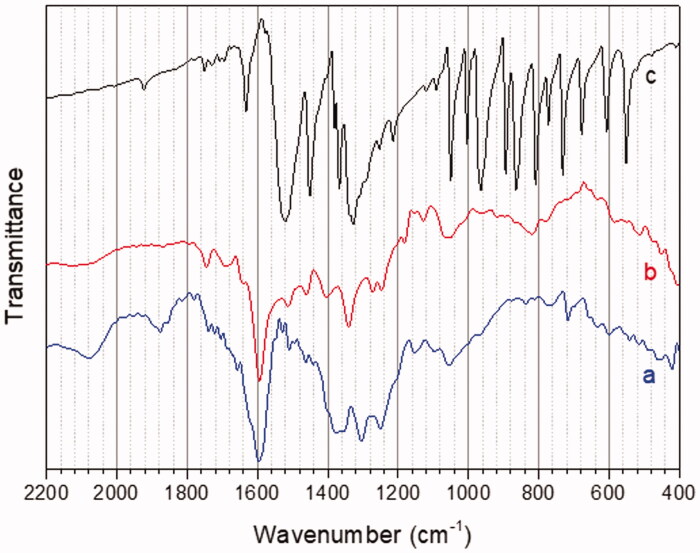
FTIR spectra of samples **2** (a), **1** (b) and of 7-nitrobenzofurazan (c).

According to literature^48^, in the infra-red spectrum of Ptb, features located at 1600, 1585, 1514, 1458, and 1353 cm^−1^ are mainly related to aromatic and olefinic stretching vibrations. Similar features can be detected in the spectra of samples **1** and **2** (lines b and a) (Figure 4).

The IR spectrum of 7-nitrobenzofurazan (line c) shows two main features at 1500 × 1360 cm^−1^, related to asymmetric and symmetric stretching of the nitro group; the same peaks can be evidenced in the spectrum of sample **1**, evidencing successful coupling of Ptb to 7-nitrobenzofurazan.

In synthesis, NEXAFS and FTIR results confirm the molecular structures proposed in Figure 1 for compounds **1** and **2**.

### 2.3. Biocompatibility of pterostilbene derivatives

**Figure 5.**
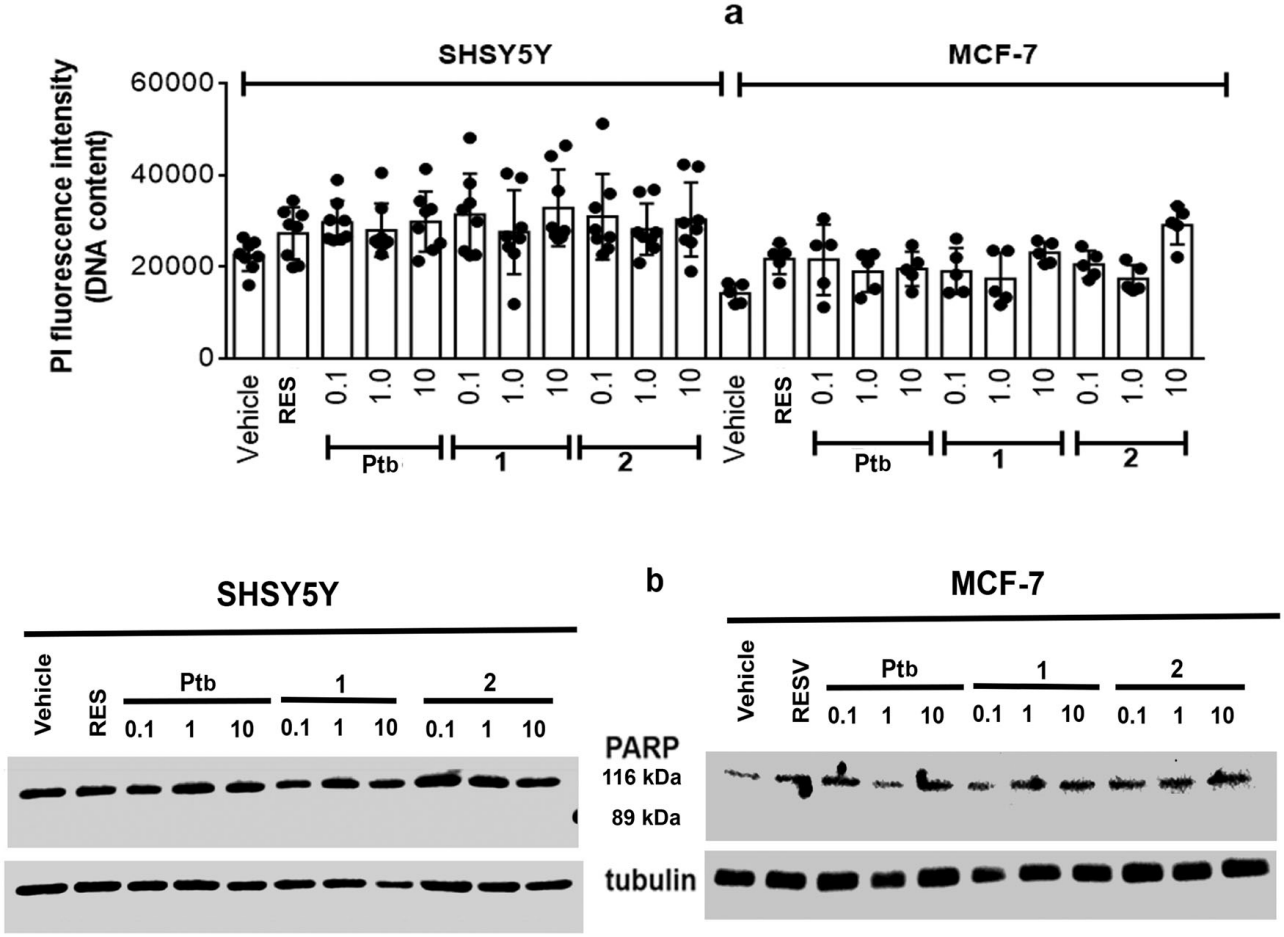
Analyses of cellular DNA content obtained from propidium iodide assay (PI) in SH-SY5Y neuroblastoma (left) and MCF-7 breast cancer (right) cells. (a) Cells were treated for 48 h with either vehicle (ethanol/DMEM, 1/10) or resveratrol (Res, 1 µM) or with different Pterostilbene (Ptb) or its fluorescent derivatives (**1** and **2**) concentrations. Data are means ± SD of height (SH-SY5Y) or five (MCF-7) different experiments. (b) Typical Western blot of five different experiments of PARP-1 (uncleaved 116 kDa, cleaved 89 kDa) in cells treated as in (a).

The putative cytotoxic effects of Ptb fluorescent derivatives was evaluated by the Propidium iodide (PI) assay, which is membrane impermeant and generally excluded from viable cells. This assay is commonly used for identifying dead cells in a population. In particular, the breast cancer (i.e. MCF-7) and the neuroblastoma (i.e. SH-SY5Y) cell lines have been used as experimental models and results were compared with that of Res 1 µM, which shares many biosynthetic pathways with pterostilbene. Notably, none of tested concentrations (i.e. 0.1–10.0 µM) of Ptb or its fluorescent derivatives modified cell morphology (data not shown) or their vitality (Figure 5(a)). The compounds effects on cell vitality were further confirmed evaluating the activation of poly (ADP-ribose) polymerase-1 (PARP-1) cleavage (Figure 5(b)). PARP-1, a DNA-binding enzyme involved in DNA repair, is a hallmark of the process of programmed cell death or apoptosis^49^. Neither Ptb nor its derivatives (**1**, **2**) modify cell vitality or promote PARP-1 cleavage confirming the very low toxicity of this compound as already reported^29^^,^^33^. Of note, none of Ptb derivatives reduces the cell number nor activates the apoptotic cascade, sustaining the inability of these compounds to trigger a stress-dependent signal transduction pathway.

**Figure 6.**
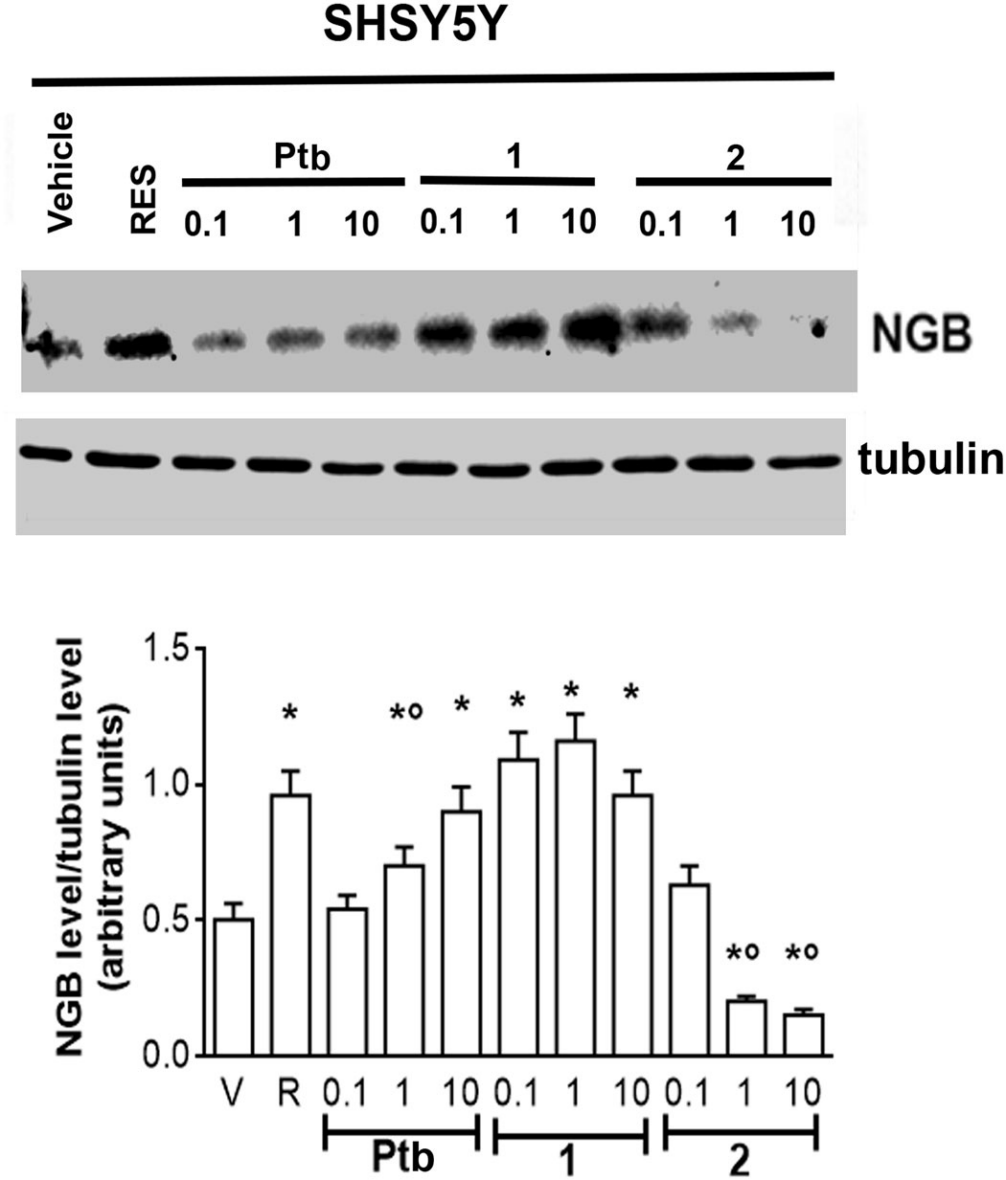
Effect of pterostilbene (Ptb) and its fluorescent derivatives (**1** and **2**) on neuroglobin (NGB) levels in neuronal-derived cells. Western blot (upper panel) and densitometric analyses (bottom panel) of NGB levels in SH-SY5Y cells. Cells were treated for 24 h with resveratrol (R, 1 µM) or with different compound concentrations. The amount of NGB was normalised by comparison with α-tubulin levels. Data are the mean ± SD of five different experiments. *p* < 0.001 was determined with ANOVA followed by Bonferroni test with respect to the vehicle (*) or to R-treated samples (°).

The lack of any toxics effects of Ptb fluorescent derivatives (**1**, **2**) raised the question if these compounds still maintain some of neuro-protective effects ascribed to their precursor^50^^,^^51^. The discovery that high levels of neuroglobin (NGB), an endogenous neuroprotective globin, are active against several brain injuries, including neurodegeneration, hypoxia, ischaemia, toxicity, and nutrient deprivation prompted us to evaluate the effect of different concentrations of Ptb and their fluorescent derivatives in SH-SY5Y cells. Res 1 µM, which increased NGB levels in neuronal derived cells reducing globin levels in breast cancer cells^52^, was used as positive control. As expected, Res increased NGB levels by 58% ± 0.6, Ptb 10 µM shows the same efficacy than Res (1 µM) although it is less effective increasing NGB levels by 30% ± 0.4. The compound **1** maintained the same efficacy than Res, with a value higher with respect to its precursor (Ptb), whereas the compound **2** reduced NGB levels under the control value. As a whole, these data, for the first time, indicate that Ptb neuroprotective effects, like Res, could be mediated by the accumulation of NGB suggesting that both polyphenols could share the same signalling pathways. Moreover, only Ptb functionalised on phenolic ring with benzofurazan (compound **1**) maintained the same efficacy of its precursor in enhancing NGB levels, while Ptb derivative containing rhodamine B-isothiocyanate (compound **2**) significantly reduced NGB levels under the control values, Figure 6.

Nowadays, NGB is regarded as an endogenous neuroprotective haeme-protein being active against several brain injuries, including hypoxia, ischaemia, toxicity, and O_2_/nutrient deprivation. The correlation between NGB expression and protection against nervous system pathologies has been demonstrated in cell systems, *in vivo* models of ND, and in human beings. Aside from the nature of the insult considered, NGB protective effects seem to depend on its overexpression and mitochondrial localisation^53^. Thus, it is not surprising that research in the field of neurodegeneration focuses on NGB inducers^14^. Among different NGB inducers, 17β-oestradiol (E2) deserves a particular attention as physiological concentrations of this hormone induce a 300% increase of NGB levels in human SKN-BE neuroblastoma cell line, mouse hippocampal neurons, primary cortical astrocytes, and striatal neurons via oestrogen receptor (ER) β-activated rapid and transcriptional events. Moreover, ligands of the subtype β of oestrogen receptor (ERβ) (e.g. Res and Ptb^54^) enhance the endogenous NGB levels facilitating neuron resilience to the stress^52^. Our data sustain that, like Res, Ptb, and its benzofurazan derivative (compound **1**, see Figure 1) could activate the ERβ pathway in SH-SY5Y neurons, while the Ptb derivative containing rhodamine B-isothiocyanate (compound **2**, see Figure 1) seems to be unable to activate the ERβ. The different biological profiles between **1** and **2** might be attributed to the great steric hindrance of rhodamine B-isothiocyanate moiety that could inhibit or impede the binding of **2** to this oestrogen receptor subtype. On the other hand, the SH-SY5Y cells, as other cells of neuronal origin, also express less levels of ERα^52^, the other oestrogen receptor subtype, which inhibition could explain the decrease of NGB levels. These data will be confirmed experimentally in the next future and our laboratory is currently very active on these issues.

The results obtained in this study highlight compound **1** as a good candidate for future studies as neuroprotective compound endowed of a good biocompatibility, absence of cellular toxicity, and the ability to be a NGB inducer as resveratrol.

## 3. Experimental section

### 3.1. Chemistry

Reagents and solvents of analytical grade were purchased from Sigma-Aldrich (St. Louis, MO) and from Fluorochem (Hadfield, UK), and were used without further purification. Chemical reactions were monitored by thin layer chromatography (TLC) using aluminium plates pre-coated with silica gel and containing a fluorescent indicator (Merck Silica Gel 60 F254). The spots on TLC were visualised by a UV lamp (254 nm). Na_2_SO_4_ was used as dehydrating agents for solutions, while they were evaporated *in vacuo* with a rotating evaporator.

Flash chromatographic purifications were performed in glass columns using silica gel 230–400 mesh Sigma-Aldrich (St. Louis, MO). Compounds were characterised by the determination of melting points and of NMR and Mass Spectrometry spectra. Melting points (m.p.) were measured with a *Leica Galen III* microscope. ^1^H and ^13 ^C NMR spectra were recorded in the opportune solvent with a Bruker Ultrashield™ 400 MHz spectrometer (Fallander, Switzerland), at 25 °C. Chemical shifts (*δ*) are given in ppm and coupling constants *(J)* are reported in Hz. Signals in NMR spectra are indicated by the following abbreviations: *s* = singlet, *d* = doublet*, t* = triplet, *q* = quartette*, m* = multiplet, *bs* = broad singlet. Mass spectra (ESI-MS) were recorded with a high-resolution Q Exactive plus Orbitrap spectrometer (Thermo Fisher Scientific, Waltham, MA), resolution 140,000 at *m*/*z* 200.

#### 3.1.1. Synthesis of (E)-tert-butyl (2-(4–(3,5-dimethoxystyryl)phenoxy)ethyl)carbamate (5)

To a solution of 4-(3,5-dimethoxystyril)phenol **3** (0.91 mmol, 1 Eq.) and K_2_CO_3_ (1.82 mmol, 2 Eq.) in anhydrous DMF (2 ml) and was added dropwise, under nitrogen, a solution of N-Boc-ethylamine bromide **4** (0.91 mmol, 1 Eq.) in anhydrous DMF. The reaction mixture was stirred at 50 °C for 8 h and then left to r.t. for 36 h monitoring by TLC. Then H_2_O was added and the aqueous solution was extracted with CHCl_3_. The organic phase was washed twice with H_2_O and ice, dehydrated with Na_2_SO_4_ and finally evaporated under reduced pressure in rotating evaporator. Compound **5** was obtained as a transparent oil (95% yield). ^1^H NMR (400 MHz, CDCl_3_) *δ*: 8.01 (s, 1H, NH); 7.44–7.41 (d, 2H, *J* = 8.7 Hz, Ar); 7.04–7.00 (d, 1H, *J* = 16.2 Hz, CH=CH); 6.91–6.87 (d, 1H, *J* = 16.2 Hz, CH = CH); 6.89–6.83 (m, 2H, Ar); 6.64–6.63 (d, 2H, *J* = 2.2 Hz, Ar); 6.37–6.36 (t, 1H, *J* = 2.2 Hz, Ar); 4.04–4.01 (t, 2H, *J* = 5.0 Hz, CH_2_); 3.82 (s, 6H, OCH_3_); 3.54–3.52 (t, 2H, *J* = 5.0 Hz, CH_2_); 1.45 (s, 9H, t-Bu).

#### 3.1.2. Synthesis of (E)-(4-(3,5-dimethoxystyryl)phenoxy)methanamine hydrochloride (6)

To a solution of compound **5** (0.82 mmol, 1 Eq.) in DCM (10 ml), was added dropwise TFA (5.74 mmol, 7 Eq.). The reaction mixture was left stirring overnight at r.t. The reaction mixture was then neutralised with saturated solution of NaHCO_3_ to pH ∼ 8. The aqueous solution was extracted with DCM and the organic phase was washed twice with H_2_O. The organic phase was dried with Na_2_SO_4_ and evaporated under reduced pressure. To the pale-yellow oil obtained was added a solution of Et_2_O • HCl, dropwise at 0 °C, to get the hydrochloride salt and then solution was evaporated. The crude product was purified by trituration in Et_2_O to afford compound **6** as a pink solid (61% yield). ^1^H NMR (400 MHz, CD_3_OD-*d_4_*) *δ*: 7.04–7.02 (d, 2H, *J* = 8.7 Hz, Ar); 6.64–6.59 (d, 1H, *J* = 16.3 Hz, CH=CH); 6.53–6.51 (d, 2H, *J* = 8.7 Hz, Ar); 6.51–6.47 (d, 1H, *J* = 16.3 Hz, CH = CH); 6.20–6.19 (d, 2H, *J* = 2.2 Hz, Ar); 5.89–5.88 (t, 1H, *J* = 2.2 Hz, Ar); 3.77–3.74 (t, 2H, *J* = 5.0 Hz, CH_2_); 3.31 (s, 6H, OCH_3_); 2.89–2.87 (t, 2H, *J* = 5.0 Hz, CH_2_).

#### 3.1.3. Synthesis of (E)-N-(2-(4-(3,5-dimethoxystyryl)phenoxy)ethyl)-7-nitrobenzo[c][1,2,5]oxadiazol-4-amine (1)

Compound **6** (0.60 mmol, 1 Eq.) and commercially available 4-chloro-7-nitrobenzofurazan **7** (0.60 mmol, 1 Eq.) were dissolved in a H_2_O/THF solution. To the resulting solution was added LiOH (0.66 mmol, 1.1 Eq.) and then the mixture was stirred at r.t. overnight, protected from light. The brown solid precipitate was washed with H_2_O and filtered under vacuum. The crude product was purified by flash chromatography (*n*-hexane/EtOAc in ratio 1.5:1) and then triturated in *n*-hexane. Compound **1** was obtained as a brown solid (93% yield). M.p.: 166–168 °C. ^1^H NMR (400 MHz, DMSO-*d_6_*) *δ*: 9.61 (s, 1H, NH); 8.56–8.54 (d, 1H, *J* = 8.7 Hz, Ar); 7.53–7.51 (d, 2H, *J* = 8.7 Hz, Ar); 7.22–7.18 (d, 1H, *J* = 16.4 Hz, CH=CH); 7.03–6.99 (d, 1H, *J* = 16.4 Hz, CH = CH); 6.97–6.95 (d, 2H, *J* = 8.7 Hz, Ar); 6.73–6.72 (d, 2H, *J* = 2.0 Hz, Ar); 6.58–6.55 (d, 1H, *J* = 8.7 Hz, Ar); 6.38–6.37 (d, 1H, *J* = 2.0 Hz, Ar); 4.33–4.31 (t, 2H, OCH_2_); 3.90 (bs, 2H, CH_2_-NH); 3.76 (s, 6H, OCH_3_). ^13 ^C NMR (100 MHz, DMSO-*d_6_*) *δ*: 159.8 (2 C, Ar-OCH_3_); 157.9 (1 C, Ar); 145.3, 144.4 (2 C, C = N); 139.3; 137.9 (2 C, Ar); 129.9 (2 C, CH = CH); 128.4, 127.8 (3 C, Ar); 126.3 (1 C, Ar-NO_2_); 121.1; 114.7; 104.1; 99.7 (7 C, Ar); 65.4 (1 C, CH_2_); 55.1 (2 C, OCH_3_); 43.0 (1 C, CH_2_). *m*/*z* ESI-MS: [M-H]^−^ 461.15.

#### 3.1.4. Synthesis of (E)-2-(3,6-bis(diethylamino)-4aH-xanthen-9-yl)-5–(3-(2–(4-(3,5-dimethoxystyryl)phenoxy)ethyl)thioureido)benzoic acid (2)

Compound **6** (0.19 mmol, 1 Eq.), as hydrochloride salt, was precipitated in 10 ml of NaOH 0.1 M. The resulting precipitate was solubilised in 5 ml of DMF, and then Rhd B-Itc **8** (0.19 mmol, 1Eq.) was added to the solution. The reaction mixture was left stirring 48 h at r.t. and protected from light. The reaction was monitored by TLC. The aqueous solution was extracted with EtOAc and then the organic layer was washed with H_2_O. The organic phase was dried over Na_2_SO_4_ and concentrated *in vacuo*. The crude product was purified by flash chromatography (CH_2_Cl_2_/MeOH, 9.5:0.5 → 9:1) to afford compound **2** as a red solid (12% yield). M.p. 140–142 °C. ^1^H NMR (400 MHz, CD_3_OD-*d_4_*) *δ*: 7.96 (s, 1H, Ar); 7.82–7.80 (m, 1H, Ar); 7.46–7.44 (d, 2H, *J* = 8.6 Hz, Ar); 7.36–7.33 (d, 2H, *J* = 9.5 Hz, Ar); 7.18–7.16 (d, 1H, *J* = 8.6 Hz, Ar); 7.07–7.03 (d, 1H, *J* = 16.2 Hz, CH=CH); 6.99–6.88 (m, 5H, Ar); 6.98–6.95 (d, 2H, *J* = 9.5 Hz, Ar); 6.92–6.88 (d, 1H, *J* = 16.2 Hz, CH = CH); 6.64–6.63 (d, 2H, *J* = 2.2 Hz, Ar); 6.36–6.35 (t, 1H, *J* = 2.2 Hz, Ar); 4.28–4.25 (t, 2H, *J* = 5.4 Hz, CH_2_); 4.06–4.03 (t, 2H, *J* = 5.4 Hz, CH_2_); 3.79 (s, 6H, OCH_3_); 3.66–3.61 (q, 8H, CH_2_); 1.30–1.26 (m, 12H, CH_3_). ^13 ^C NMR (100 MHz, MeOD-*d_4_*) *δ*: 183.0 (1 C, C = S); 163.4 (1 C, COOH); 162.7 (2 C, Ar-OCH_3_); 160.2 (1 C, Ar-OCH_2_); 159.6, 157.0, 141.3, 133.5, 132.0, 131.4, 130.0 (11 C, Ar); 129.1 (2 C, CH = CH); 128.0, 116.2, 115.4, 115.0, 105.5, 100.7, 97.2 (17 C, Ar); 67.5 (1 C, OCH_2_); 56.0 (2 C, OCH_3_); 46.9 (4 C, CH_2_); 45.4 (1 C, CH_2_); 33.3, 30.9, 23.9; 14.6, 13.0 (4 C, CH_3_). *m/z* ESI-MS: [M + H]^+^ 799.35.

### 3.2. Optical and structural characterisations

UV–visible absorption measurements were carried out in the range 200–800 nm, using DMSO solution in quartz cells, by Shimadzu 2401 PC UV–vis spectrophotometer^55^. Fluorescence measurements were carried out, using DMSO solution in quartz cells, by Varian–Carey Eclipse Fluorescence Spectrophotometer.

Fourier transform infra-red spectroscopy (FT-IR) spectra have been recorded in the 4000–400 cm^−1^ range with a Bruker Vector 22 equipped with a DTGS detector. Thin films of samples **1** and **2**, deposited from DMSO solution onto gold substrates, were analysed in reflectance mode with a Specac P/N 19650 series monolayer/grazing angle accessory. KBr pellets of 7-nitrobenzofurazan samples were prepared to record the FTIR spectrum.

Near-edge X-ray absorption fine structure (NEXAFS) spectra were acquired at the BEAR beamline (bending magnet for emission absorption and reflectivity) at the ELETTRA storage ring. BEAR is installed at the left exit of the 8.1 bending magnet exit. The apparatus is based on a bending magnet as a source and beamline optics delivering photons from 5 eV up to about 1600 eV with selectable degree of ellipticity. The UHV end station is equipped with a movable hemispherical electron analyser and a set of photodiodes to collect angle-resolved photoemission spectra, optical reflectivity, and fluorescence yield. In these experiments, we used ammeters to measure drain current from the sample. C, N, and O K-edge spectra were collected at grazing (20°) incidence angles of the linearly polarised photon beam with respect to the sample surface. In addition, our carbon, nitrogen and oxygen K-edge spectra have been further calibrated using the resonance at 285.00 eV, assigned to the C = C aromatic 1 s − π* transition, the resonance at 401.00 eV, assigned to the 1 s − π* transition of the C_ar_-N bond and the resonance at 531.5 eV, assigned to the C = O carbonyl 1 s − π* transition, respectively. The raw C, N, and O K-edge NEXAFS spectra were normalised to the incident photon flux by dividing the sample spectrum by the spectrum collected on a freshly sputtered gold surface. Spectra were then normalised subtracting a straight line that fits the part of the spectrum below the edge and assessing to 1 the value at 330.00, 430.00, and 560.00 eV for carbon, nitrogen, and oxygen, respectively.

### 3.3. Cell lines and *in vitro* assays

#### 3.3.1. Cell culture and stimulation

Human breast cancer cells, MCF‐7, and human neuroblastoma cells, SH-SY5Y (ATTC, LGC Standards S.r.l., Milano, Italy) were grown in air containing 5% CO_2_ in modified, phenol red‐free, Dulbecco’s Modified Eagle’s Medium (DMEM) medium. Ten % (vol/vol) of charcoal‐stripped foetal calf serum, l‐glutamine (2 mM), gentamicin (0.1 mg/mL), and penicillin (100 U/mL) were added to the media before use. Cells were used at passage 13–17. The cell line authentication was periodically performed by amplification of multiple short tandem repeat loci by BMR genomics S.r.l (Padova, Italy). Cells were treated for 48 h with either vehicle (ethanol [EtOH]/DMEM, 1:10; vol/vol) or resveratrol (Res, 1 µM) or with different concentration of Pterostilbene and its fluorescent derivatives **1** and **2**.

#### 3.3.2. Western blot assay

Briefly, after the treatments, cells were lysed and protein were solubilised in the YY buffer (50 mM HEPES at pH 7.5, 10% glycerol, 150 mM NaCl, 1% Triton X‐100, 1 mM EDTA, and 1 mM EGTA) containing 0.70% (wt/vol) sodium dodecyl sulphate (SDS). Total proteins were quantified using the Bradford protein assay (Bio‐Rad Laboratories, Hercules, CA). Solubilised proteins (20 µg) were resolved by 7% or 15% SDS‐polyacrylamide gel electrophoresis at 100 V for 1 h at 24.0 °C and then transferred to nitrocellulose with the Trans‐Blot Turbo Transfer System (Bio‐Rad Laboratories, Hercules, CA) for 10 min. The nitrocellulose was treated with 5% (wt/vol) bovine serum albumin in 138.0 mM NaCl, 25.0 mM Tris, pH 8.0, at 24.0 °C for 1 h and then probed overnight at 4.0 °C with anti‐PARP‐1 (final dilution, 1:1000) or with anti-NGB (final dilution, 1:1000) antibodies. Moreover, anti‐α‐tubulin (final dilution, 1:30,000) antibody was used to normalise the protein loaded. The antibody reaction was visualised with the chemiluminescence western blot analysis detection reagent (Amersham Biosciences, Little Chalfont, UK). The densitometric analyses were performed by the ImageJ software for Microsoft Windows (National Institute of Health, Bethesda, MD).

#### 3.3.3. Propidium iodide (PI) assay

After grown up to 80% confluence in 96‐well plate, cells were stimulated as reported above. Fixation and permeabilization were performed through ice cold EtOH 70% for 15 min at −20 °C. After EtOH solution removal, the cells were incubated with PI buffer for 30 min in the dark. Again, solution removal was performed, and the cells were rinsed with PBS solution. The fluorescence was revealed (excitation wavelength: 537 nm; emission wavelength: 621 nm) with TECAN Spark 20 M multimode microplate reader (Life Science, Padua, Italy).

#### 3.3.4. Statistical analysis

The statistical analyses were performed by Student’s *t*-test to compare two sets of data or by ANOVA followed by Bonferroni test to compare different group of data by INSTAT software system for Windows. In all cases, *p* < 0.05 was considered significant.

## Supplementary Material

Supplemental Material
