# Supplementary material for: Pterostilbene fluorescent probes as potential tools for targeting neurodegeneration in biological applications
Source: J Enzyme Inhib Med Chem. 2022 Jun 27;37(1):1812–20. doi: 10.1080/14756366.2022.2091556 (PMC9246042; doi:10.1080/14756366.2022.2091556)
Supplement: Supplemental Material [file IENZ_A_2091556_SM5515.pdf]

Pterostilbene fluorescent probes as potential tools for targeting neurodegeneration in biological applications

Lidia Ciccone[a,b], Susanna Nencetti[a,b,*] Maria Marino[c], Chiara Battocchio[c], Giovanna Iucci[c], Iole Venditti[c], Martina Marsotto[c], Emiliano Montalesi[c], Simone Socci[d], Beatrice Bargagna[d] and Elisabetta Orlandini [d,e,*]

[a] Department of Pharmacy University of Pisa, Via Bonanno 6 -56126- Pisa (IT); [b] CISUP - Centre for Instrumentation Sharing - University of Pisa, [c] Department of Science, University Roma Tre, Viale Marconi 446 - 00146 - Rome (IT); [d] Department of Earth Science University of Pisa, via Santa Maria, 53, 56126, Pisa (IT); [e] Research Centre E.Piaggio, University of Pisa, Pisa 56122, Italy

*corresponding authors *Elisabetta Orlandini, Department of Earth Science University of Pisa, via Santa Maria, 53, 56126, Pisa (IT), elisabetta.orlandini@unipi.it; Susanna Nencetti, Department of Pharmacy University of Pisa, Via Bonanno 6 -56126- Pisa (IT), susanna.nencetti@unipi.it*

Table of Contents

^{1}H and ^{13}C NMR (DMSO-*d*$_6$) spectra of compound **1** page 2

ESI-MS: $[M\text{-}H]^-$ spectra of compound **1** page 3

^{1}H and ^{13}C NMR (CD$_3$OD-*d*$_4$) spectra of compound **2** page 4

ESI-MS: $[M\text{-}H]^-$ spectra of compound **2** page 5

Figure S1 and **Table S1** optical characterization of NBD page 6

Figure S2 and **Table S2** optical characterization of compound **1** page 7

Figure S3 and **Table S3** optical characterization of compound **2** page 8

Figure S4 and **Table S4** optical characterization of Res page 9

^{1}H and ^{13}C NMR (DMSO-d_6) spectra of compound **1**.

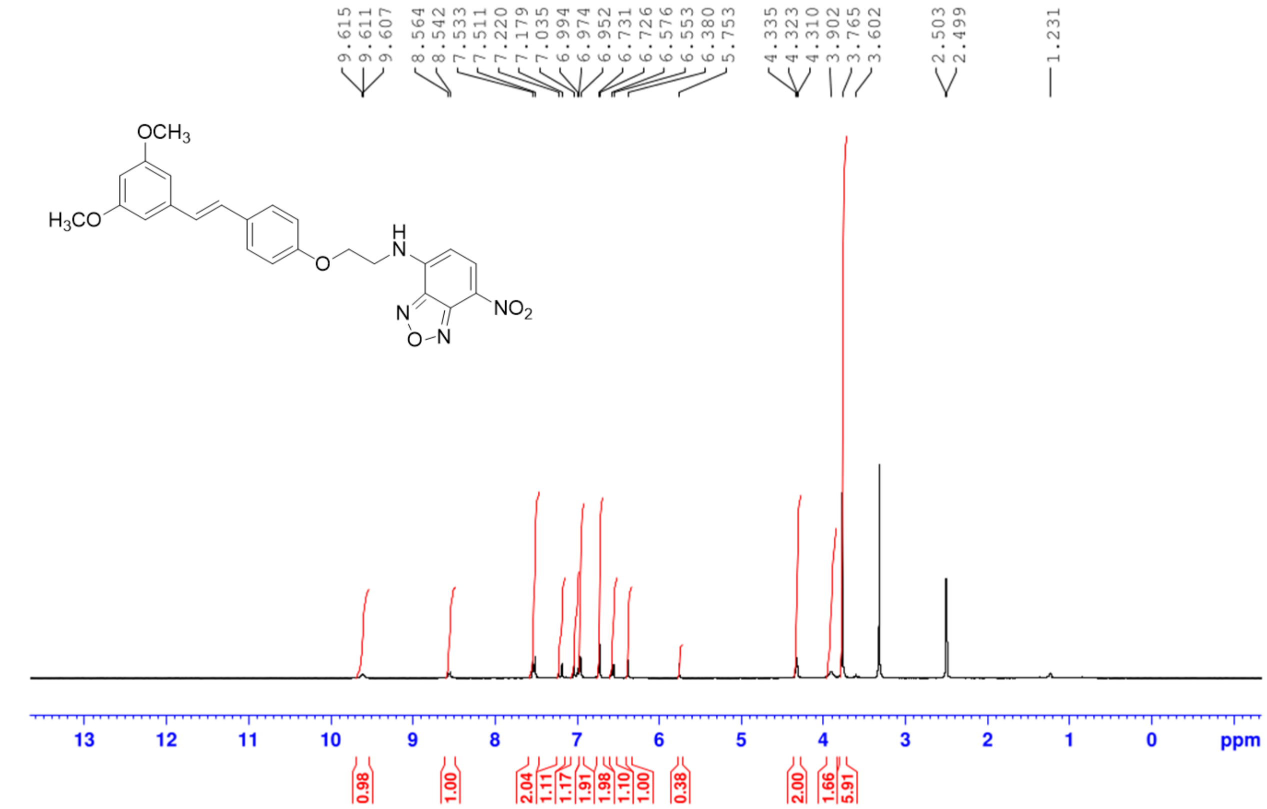

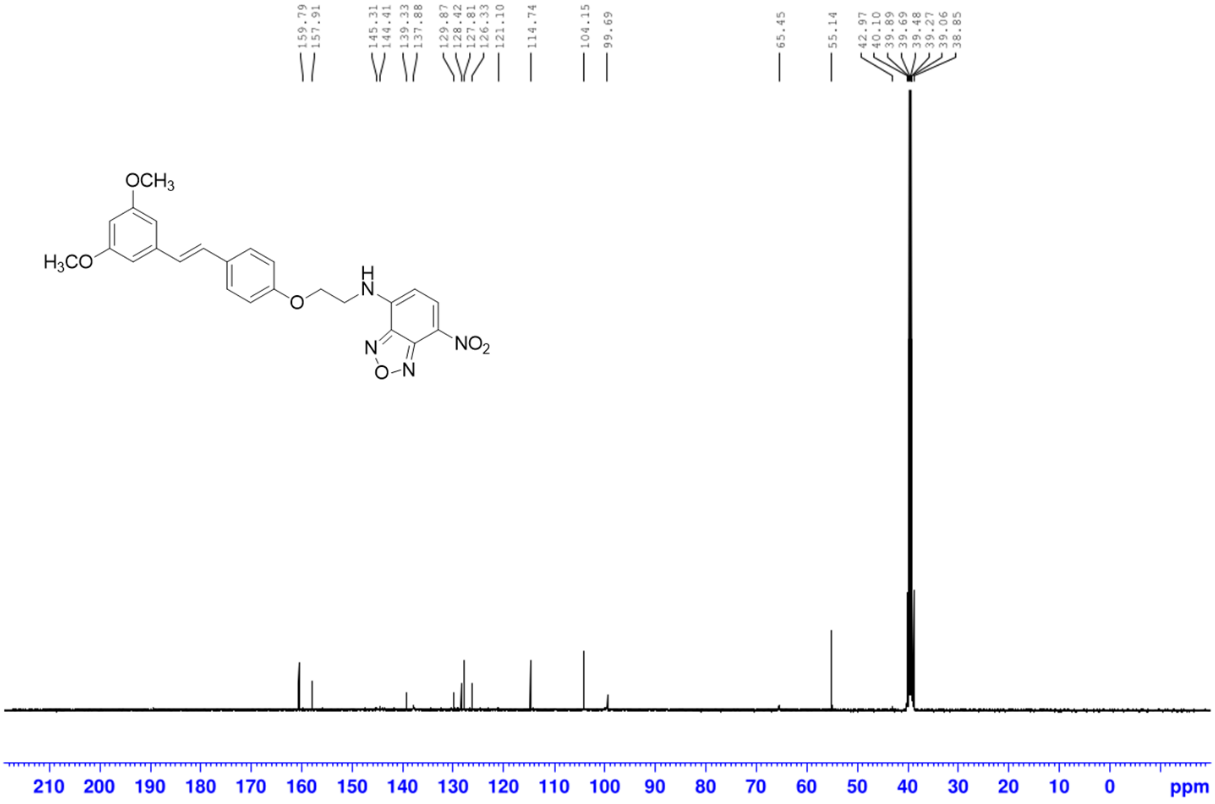

ESI-MS: [M-H]$^{-}$ compound **1**.

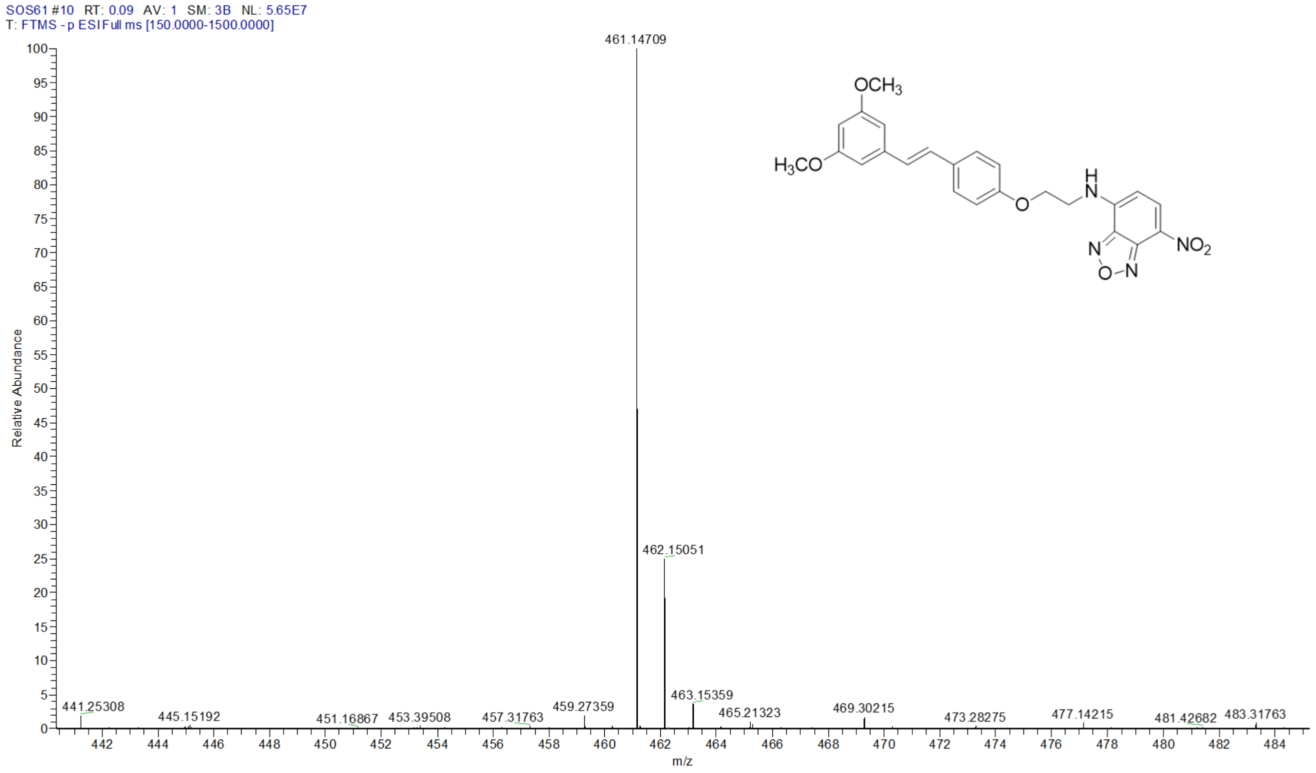

^{1}H and **^{13}C NMR (CD_3OD-*d4*)** spectra of compound **2**.

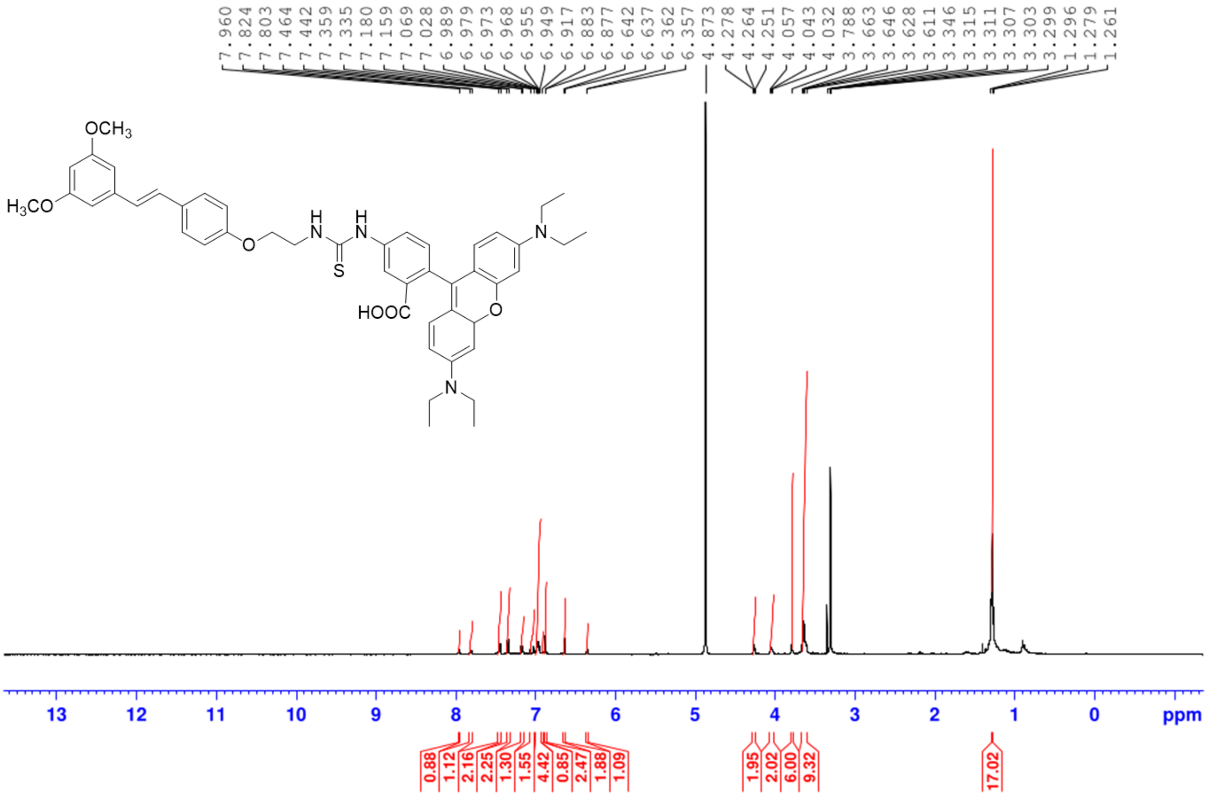

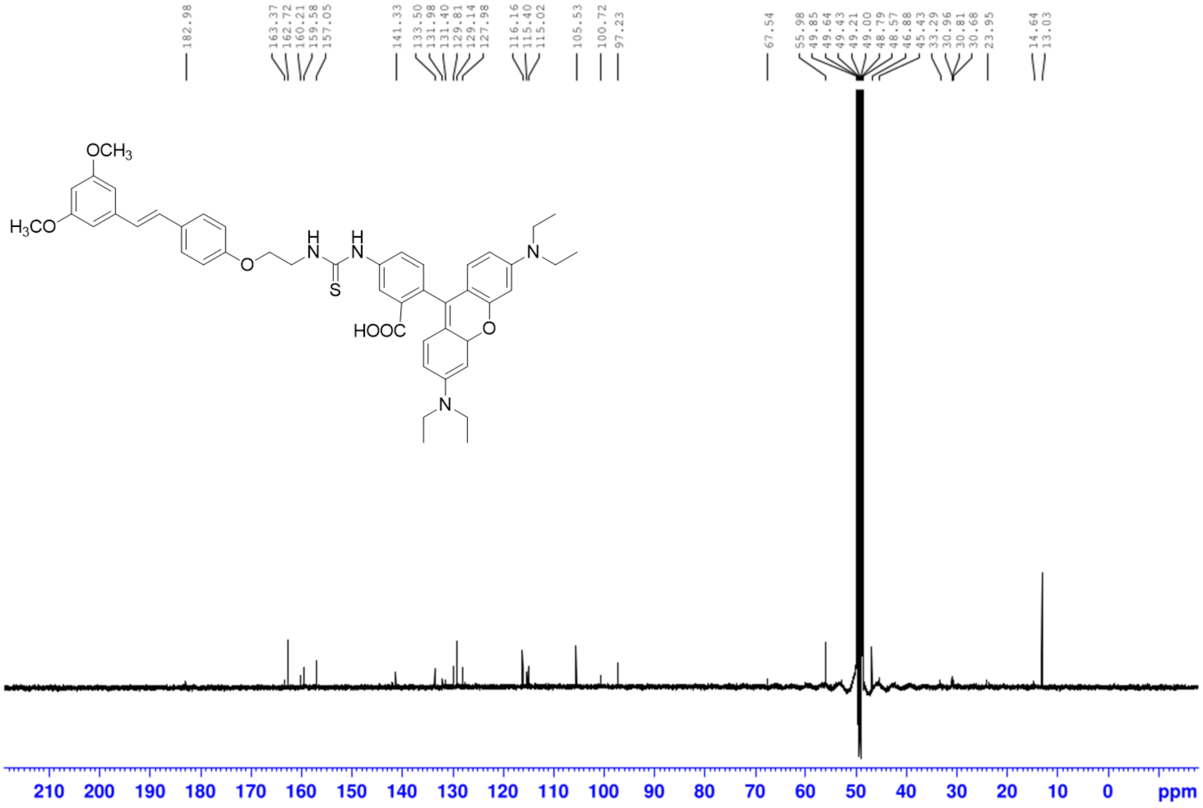

ESI-MS: [M+H]$^+$ compound **2**.

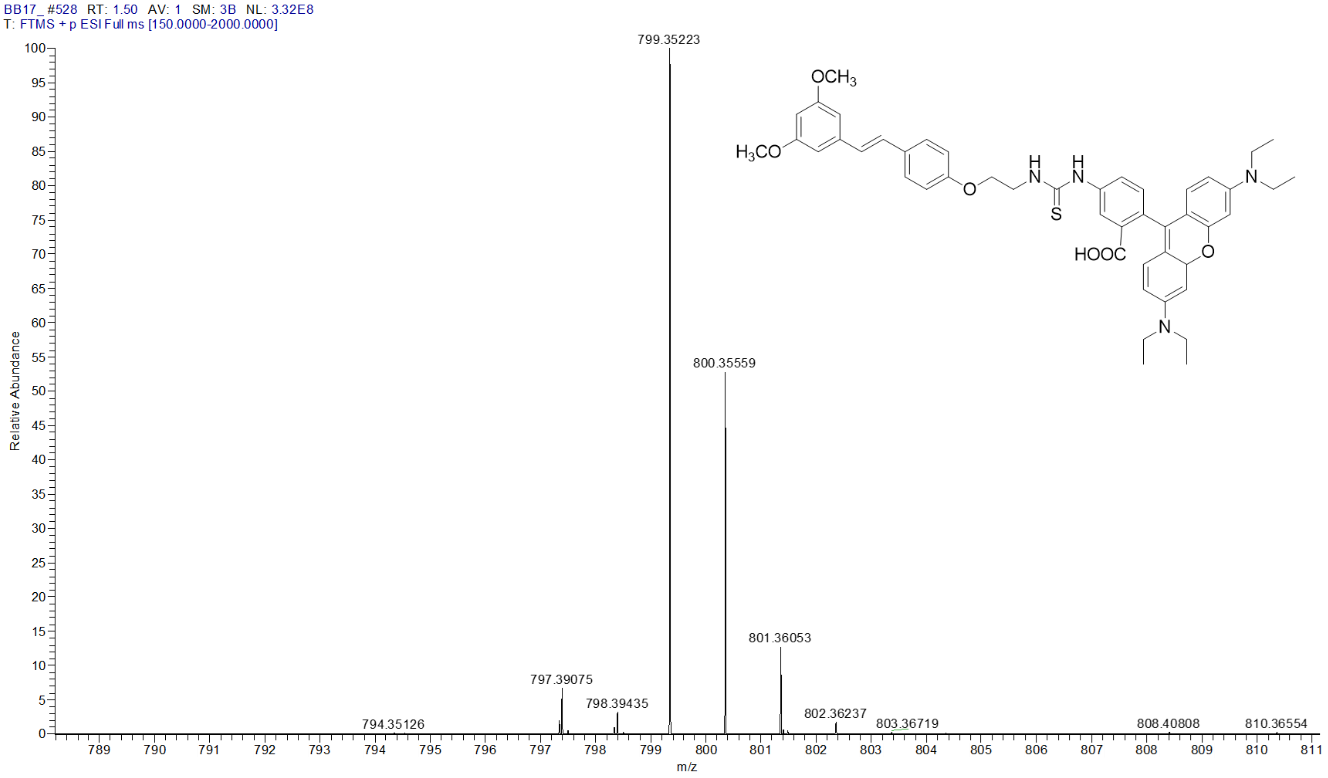

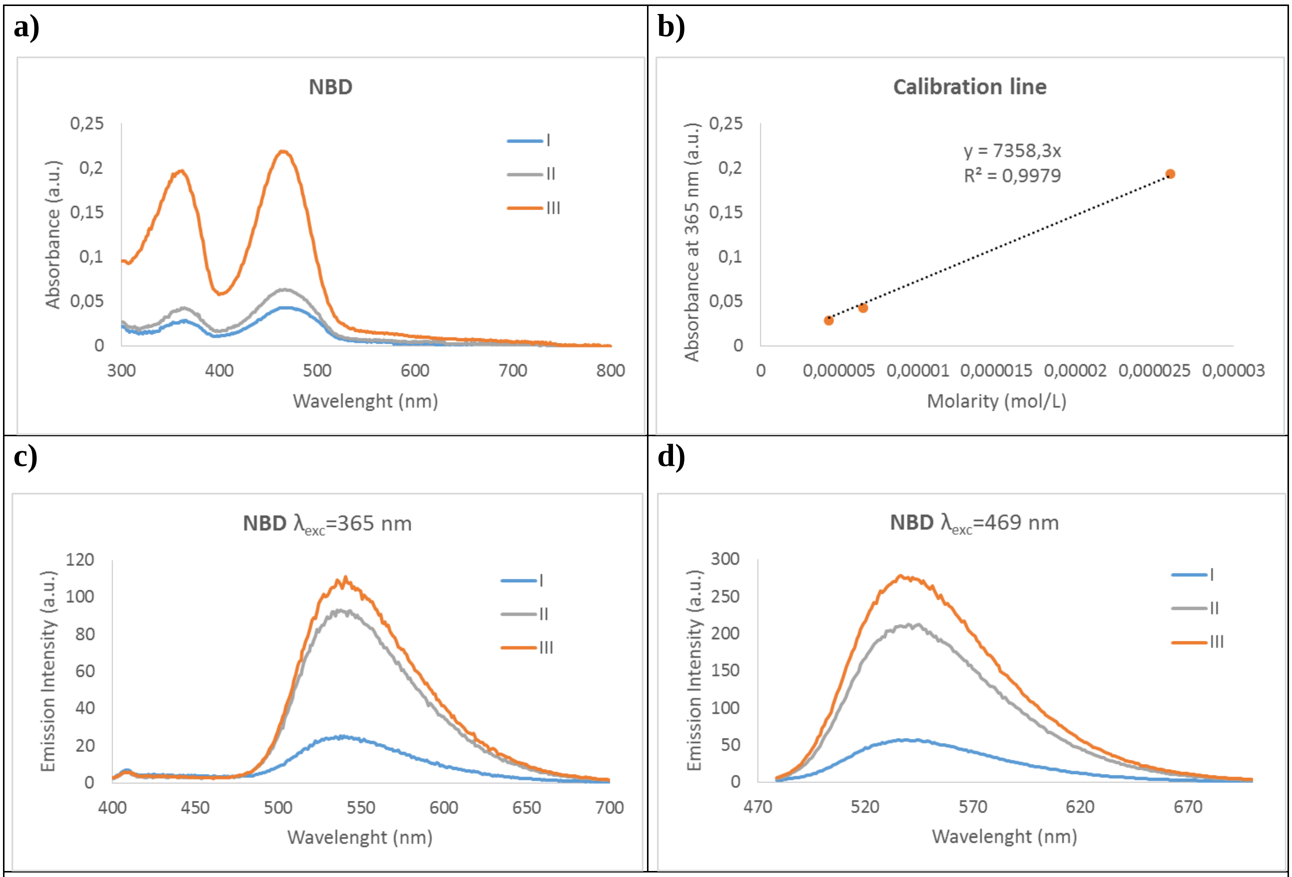

Figure S1. a) Absorption spectrum of three different concentrations of NBD; **b)** Calibration line of NBD; **c)** Emission spectrum of NBD obtained at λ_{exc} = 365 nm; **d)** Emission spectrum of NBD obtained at λ_{exc} = 469 nm.

Table S1. Concentrations of the three DMSO solutions of NBD, their absorption intensities at 365 nm and 467 nm and emission peak intensities at 541 and 547 nm. The value of the absorption coefficient ε of NBD is also reported.

**Solutions of NBD**	**Molarity (mol/L)**	**$A_{\lambda=365nm}$**	**$A_{\lambda=467nm}$**	**ε (cm^{-1} M^{-1})**	**$I_{\lambda=541nm}$**	**$I_{\lambda=547nm}$**
I	4.3E-06	0.29	0.043		24.30	54.74
II	6.5E-06	0.043	0.064	7358	92.05	206.01
III	2.6E-05	0.193	0.218		111.23	271.40

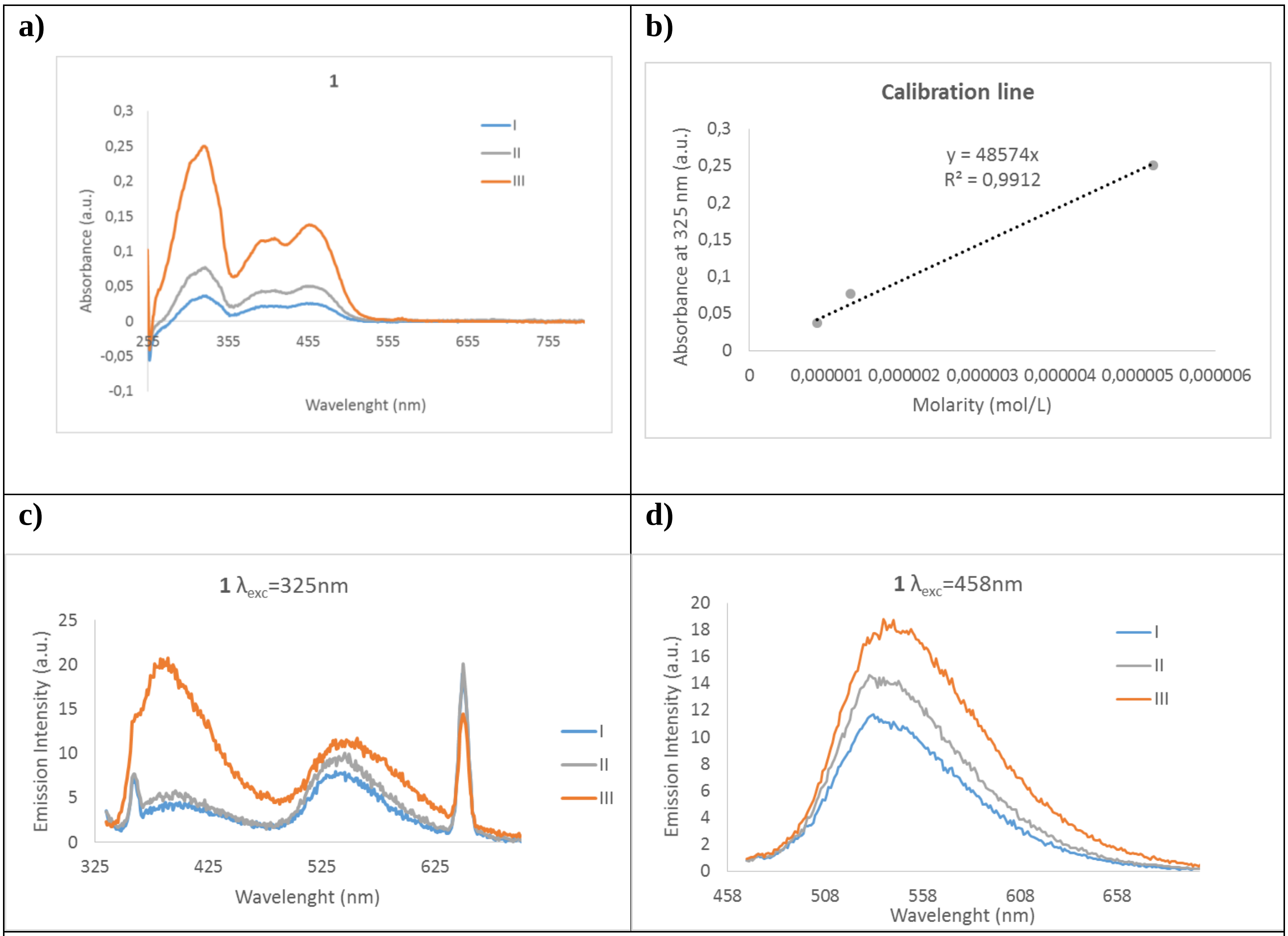

Figure S2. a) Absorption spectrum of three different concentrations of compound **1**; **b)** Calibration line of compound **1**; **c)** Emission spectrum of compound **1** obtained at λ_{exc} = 325 nm; **d)** Emission spectrum of compound **1** obtained at λ_{exc} = 458 nm.

Table S2. Concentrations of the three DMSO solutions of **1**, their absorption intensities at 325 nm and 458 nm and emission peak intensities at 387, 537 nm. The value of the absorption coefficient ε of **1** is also reported.

**Solutions of 1**	**Molarity (mol/L)**	**$A_{\lambda=325nm}$**	**$A_{\lambda=458nm}$**	**ε (cm^{-1} M^{-1})**	**$I_{\lambda=387nm;537nm}$**	**$I_{\lambda=537nm}$**
I	8.7E-07	0.037	0.026	48574	4.45;7.95	11.11
II	1.3E-06	0.077	0.051		5.14;9.50	14.43
III	5.2E-06	0.25	0.137		20.26;11.56	18.80

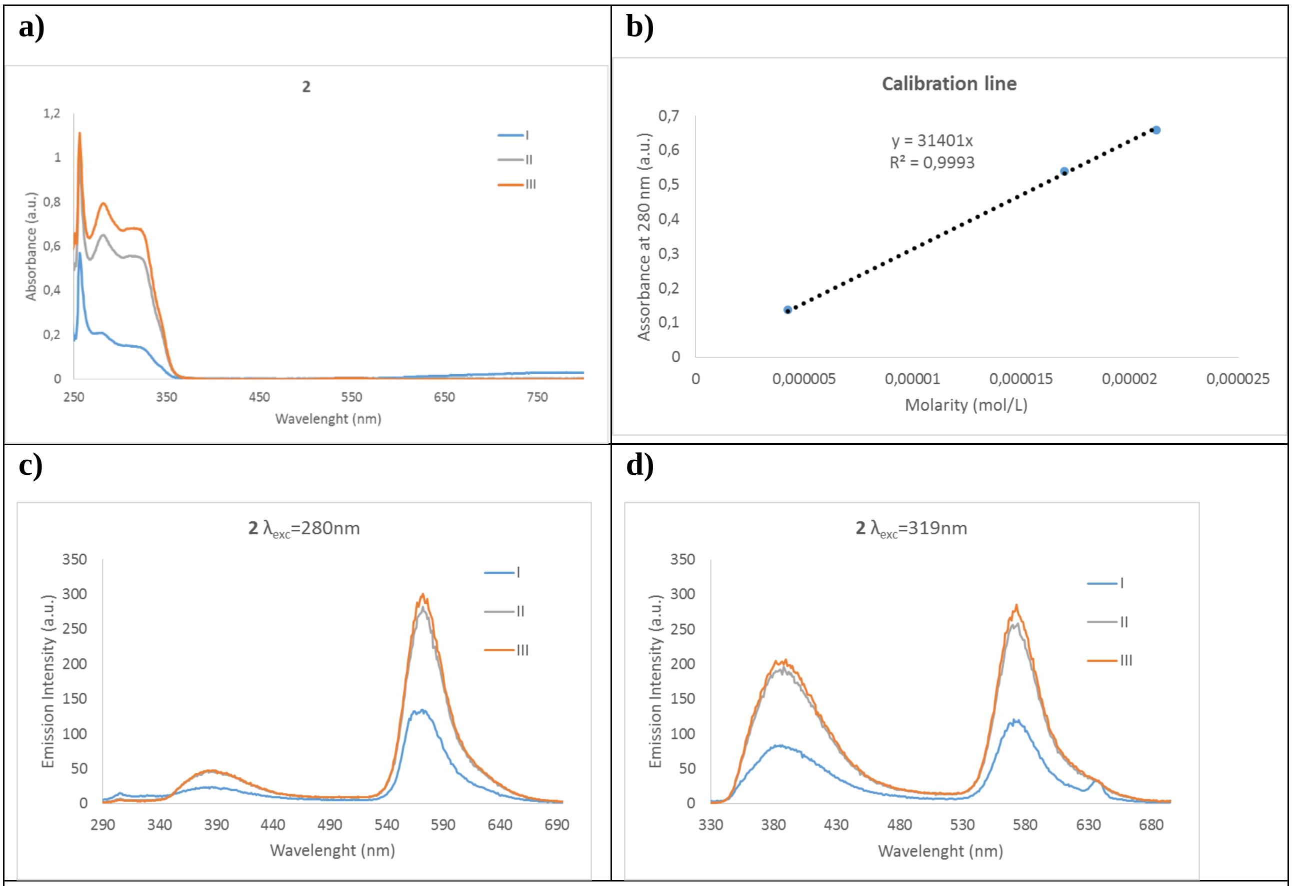

Figure S3. a) Absorption spectrum of three different concentrations of compound **2**; **b)** Calibration line of compound **2**; **c)** Emission spectrum of compound **2** obtained at λ_{exc} = 280 nm; **d)** Emission spectrum of compound **2** obtained at λ_{exc} = 319 nm.

Table S3. Concentrations of the three DMSO solutions of **2**, their absorption intensities at 280 nm and 319 nm and emission peak intensities at 388 and 575 nm. The value of the absorption coefficient ε of **2** is also reported.

**Solutions of 2**	**Molarity (mol/L)**	**$A_{\lambda=280nm}$**	**$A_{\lambda=319nm}$**	**ε (cm^{-1} M^{-1})**	**$I_{\lambda=388nm;575nm}$**	**$I_{\lambda=388nm;575nm}$**
I	4.2E-06	0.21	0.15	31401	22.50;130.87	82.17;119.82
II	1.7E-05	0.65	0.55		46.34;272.27	196.30;253.62
III	2.1E-05	0.79	0.68		47.89;287.71	200.19;270.60

a) b) c)

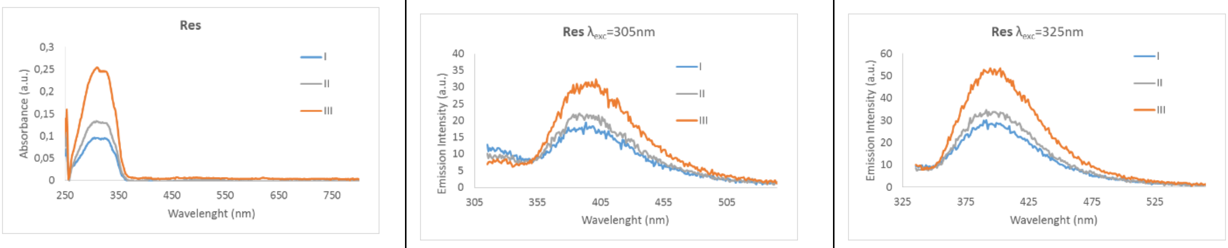

Figure S4. a) Absorption spectrum of resveratrol (Res); **b)** and **c)** Emission spectra of Res.

Table S4. Concentrations of the three DMSO solutions of Res.

**Solutions of RES**	**Molarity (mol/L)**	$\mathbf{A_{\lambda=310nm}}$	$\mathbf{A_{\lambda=319nm}}$	$\mathbf{I_{\lambda=397nm}}$	$\mathbf{I_{\lambda=397nm}}$
I	4.3E-06	0.61	0.60	53	30
II	6.5E-06	0.26	0.25	34	22
III	1.3E-05	0.13	0.13	28	18
